# Metabolomic profiling delineates the role of adenosine in oocyte quality and embryonic development

**DOI:** 10.1038/s41419-026-08886-9

**Published:** 2026-06-09

**Authors:** Jihong Yang, Hui Wang, Yangbai Li, Xinyue Zhang, Ting Feng, Suying Li, Yao Chen, Yuntian Xu, Ruizhi Feng, Minjian Chen, Yun Qian

**Affiliations:** 1 Reproductive Obstetrics and Gynecology Center, The Second Affiliated Hospital of Nanjing Medical University, Nanjing, Jiangsu China; 2 State Key Laboratory of Reproductive Medicine and Offspring Health, Department of Histology and Embryology, Nanjing Medical University, Nanjing, Jiangsu China; 3 Key Laboratory of Modern Toxicology of Ministry of Education, Nanjing Medical University, Nanjing, Jiangsu China; 4 State Key Laboratory of Reproductive Medicine and Offspring Health, Nanjing Medical University, Nanjing, Jiangsu China; 5 State Key Laboratory of Reproductive Medicine and Offspring Health, Center for Global Health, School of Public Health, Nanjing Medical University, Nanjing, Jiangsu China

**Keywords:** Embryonic induction, Embryology

## Abstract

Metabolic determinants of oocyte quality and embryonic development remain incompletely understood. Here, we profiled metabolites in human cumulus cells (CCs) and follicular fluid (FF), validated in two mouse models, and integrated transcriptomics with receptor blockade to define mechanisms. In human CCs, adenosine was higher in cycles yielding fewer high-quality embryos and discriminated embryo quality (ROC AUC = 0.75). Conversely, FF adenosine was reduced in the same context. In mice, low-quality oocytes and their associated CCs accumulated adenosine, revealing an intra- vs extracellular disequilibrium. The imbalance aligned with reduced expression of the adenosine transporters ENT1/ENT2 and the gap-junction component CX37. Functionally, supplementation with exogenous adenosine restored early embryonic development from low-quality oocytes, lowering oxidative stress and spindle/chromosome errors via adenosine receptors. Smart-seq2 transcriptomic analysis and functional experiments showed partial normalization of programs governing meiosis and cellular stress responses, including correction of CycB1/Cdc27 (MPF/APC/C) and JNK-linked pathways. Together, we identify adenosine disequilibrium as a metabolic fingerprint of poor oocyte competence and show that receptor-mediated adenosine signaling tunes MPF-related and oxidative stress pathways to rescue developmental potential. These findings provide a mechanistic and translational basis for early prediction and culture optimization in ART.

**Figure:**
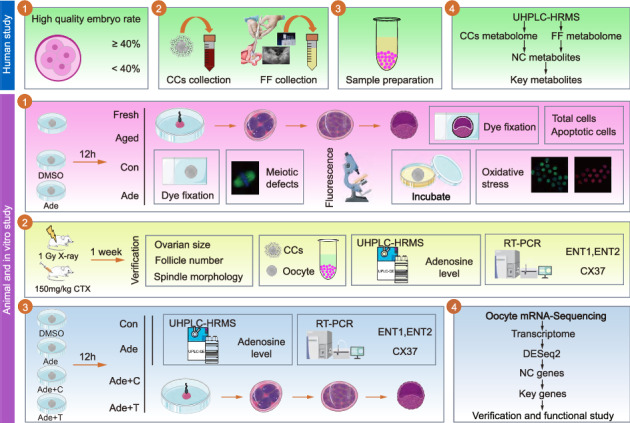

## Introduction

Infertility affects a significant proportion of individuals in their reproductive years, with approximately 10-15% of couples experiencing difficulty conceiving [1, 2]. This prevalence remains stable and imposes substantial burdens across mental, physical, sexual, and social domains, leading to notable social and economic challenges [3, 4]. Assisted reproductive technologies (ART) have provided hope to many such couples, but success depends critically on oocyte and embryo quality [5, 6]. Meanwhile, the developmental potential of in vitro maturation (IVM) oocytes has been reduced compared with those matured in vivo, underscoring the need for new additives to improve IVM competence [7]. However, reliable approaches to predict and enhance oocyte quality and embryonic developmental capacity remain limited. Understanding the molecular events related to oocyte quality and embryonic development is critical for understanding reproduction. Nowadays, metabolism has been indicated as a key molecular layer in relation to oocyte developmental competence; however, despite advancing methodologies, our understanding of how oocyte metabolic function relates to developmental potential remains incomplete [7]. Metabolomics, a non-invasive omics technology, captures the global metabolic footprint of cells and their microenvironments [8]. Metabolism is located at the end of biological regulation, making metabolomics closer to the phenotype than genomics and proteomics [9, 10]. Delineating key metabolic events offers a promising route to early assessment of oocyte and embryo quality and to uncover mechanisms underpinning developmental competence [11, 12].

An increasing number of metabolomic studies have focused on oocyte-associated metabolism, investigating metabolites present in follicular fluid (FF) and oocyte maturation culture medium [13–17]. Additionally, several analyses have been conducted on the metabolomics of granulosa cells [18–20]. Granulosa cells comprise mural granulosa cells (MGCs) and cumulus cells (CCs) [21]; the latter envelop the oocyte and supply metabolites essential for both nuclear and cytoplasmic maturation [22, 23]. Through gap junctions with CCs, the oocyte acquires the competence necessary for fertilization and early embryonic development [24], and disruptions in this molecular cooperation indicate declining oocyte quality [25, 26]. Additionally, the characteristics of mitochondria in CCs are also associated with embryo quality [27]. Given the close association between CCs and the oocyte, metabolites derived from CCs may be used as biomarkers to assess oocyte quality and predict developmental competence. Despite multiple studies attempting to correlate oocyte metabolism with ART outcomes and enhance IVM [7], there remains a need for a comprehensive, integrative metabolomic approach that simultaneously encompasses CCs, oocytes, and FF. Such an approach would provide a novel understanding of the metabolic landscape and address the knowledge gap related to critical events influencing oocyte quality and embryonic development.

In this study, we aimed to comprehensively investigate the metabolic signature associated with oocyte quality and embryonic development. To achieve this goal, we started from CCs, oocytes, and FF, systematically integrating metabolomic and transcriptomic analyses of both human and mouse models, exploring the mechanism of adenosine transmission between cells, and clarifying the key role of adenosine in regulating oocyte quality and embryonic development.

## Results

### Clinical epidemiological characteristics

**Fig. 1:**
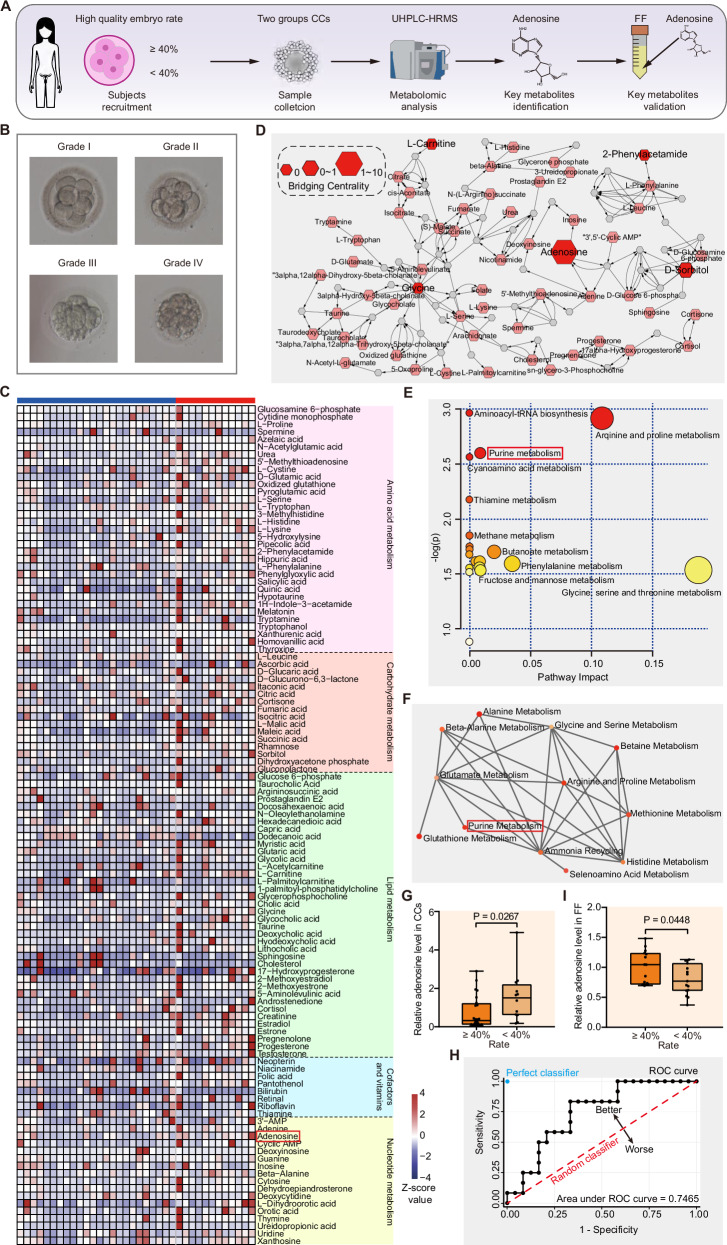
Metabolomic analysis reveals adenosine as a key metabolite in CCs and FF related to embryo quality. **A** The workflow for sample collection and metabolome profiling is depicted schematically. **B** Representative images of various grades of embryos. **C** The heatmap presents the distribution of 112 metabolites across distinct groups, categorized by high-quality embryo rate of < and ≥ 40%, within the five primary metabolic pathways: amino acid metabolism, carbohydrate metabolism, lipid metabolism, metabolism of cofactors and vitamins, and nucleotide metabolism (*n* = 12 and 24 for high-quality embryo < and ≥ 40%, respectively). Notably, adenosine exhibited an elevation in CCs within the group of high-quality embryos < 40%. **D** The interconnection of metabolites was assessed using bridging centrality, which identified adenosine as the most crucial node, signifying its significance. Metabolites demonstrating a significant association with high-quality embryos are highlighted in red, with their size indicating the extent of their bridging centrality. Other metabolites with available data are colored pink. **E** Pathway analysis revealed that purine metabolism, encompassing adenosine and glycine, was a critical pathway that exhibited alterations. **F** Enrichment analysis revealed that purine metabolism, specifically involving adenosine and glycine, was associated with significant changes in multiple metabolic pathways. **G** The comparative alterations in adenosine levels within CCs were examined between the groups of high-quality embryo rate < and ≥ 40% (*n* = 12 and 24 for high-quality embryo rate < and ≥ 40%, respectively). **H** The ROC curve analysis demonstrated that adenosine exhibited predictive ability for distinguishing between the two groups. **I** The relative adenosine level was measured in FF obtained from two distinct groups of high-quality embryo rate < and ≥ 40% (*n* = 15 for each group). Statistical analyses in **G** and **I** were performed by a two-tailed Student’s t-test, and the data in **G** and **I** are visually presented using interquartile range boxplots. The upper and lower boundaries of each box correspond to the 75th and 25th percentiles, respectively. Additionally, the median separating the inner quartiles is displayed, along with “whiskers” extending from the maximum to the minimum values.

In this study, we identified key metabolites associated with embryo quality in human subjects (Fig. 1A). The female participants (mean age, 33.333 ± 1.112 years; body mass index (BMI), 22.639 ± 0.483 kg/m^2^) had basal serum follicle-stimulating hormone (FSH) levels of 7.649 ± 0.432 IU/ml, and anti-Müllerian hormone (AMH) levels of 4.363 ± 0.528 ng/ml; male partners (mean age, 35.583 ± 1.180 years; BMI 24.493 ± 0.577 kg/m^2^) had semen volume of 2.897 ± 0.216 ml, sperm density (* 10^6^/ml) of 59.542 ± 9.599, sperm motility of 54.726 ± 4.243%, sperm deformity of 92.938 ± 0.918%, and sperm fragmentation rate of 13.369 ± 1.279%. The number of retrieved oocytes was 9.611 ± 1.320, with a fertilization rate of 71.236 ± 4.060%. Participants were stratified by a 40% high-quality embryo threshold (grades I–II) [28, 29] (Fig. 1B). No significant differences were observed between the groups in baseline clinical characteristics, except for the high-quality embryo rate. This finding underscored the need for further investigation into metabolomic differences to elucidate the underlying mechanisms responsible for this disparity (Table 1).

**Table 1:** Clinical epidemiological information of participants who provided CCs.

	All (*n* = 36)	Rate of high-quality embryo < 40% (*n* = 12)	Rate of high-quality embryo ≥ 40% (*n* = 24)	*P* value
Female factors				
Age (years)	33.333 ± 1.112	32.080 ± 2.013	33.960 ± 1.343	0.435
BMI (kg/m^2^)	22.639 ± 0.483	21.640 ± 0.907	23.160 ± 0.546	0.137
Basal serum FSH (mIU/ml)	7.649 ± 0.432	7.689 ± 0.534	7.629 ± 0.599	0.949
AMH (ng/ml)	4.363 ± 0.528	3.791 ± 0.969	4.707 ± 0.620	0.410
Male factors				
Age (years)	35.583 ± 1.180	33.670 ± 2.040	36.540 ± 1.418	0.257
BMI (kg/m^2^)	24.493 ± 0.577	25.540 ± 1.108	23.950 ± 0.650	0.195
Semen volume (ml)	2.897 ± 0.216	2.336 ± 0.285	3.165 ± 0.275	0.072
Sperm density (* 10^6^/ml)	59.542 ± 9.599	64.090 ± 20.820	57.370 ± 10.460	0.749
Sperm motility (%)	54.726 ± 4.243	51.770 ± 9.170	56.140 ± 4.622	0.637
Sperm deformity (%)	92.938 ± 0.918	92.840 ± 1.910	93.080 ± 1.159	0.916
Sperm fragmentation rate (%)	13.369 ± 1.279	11.080 ± 2.523	14.850 ± 1.273	0.154
Laboratory factors				
No. of oocyte retrieved	9.611 ± 1.320	6.167 ± 0.860	11.330 ± 1.847	0.064
Rate of fertilization (%)	71.236 ± 4.060	70.760 ± 8.464	71.480 ± 4.525	0.935
Rate of high-quality embryo (%)	55.256 ± 5.711	15.228 ± 4.697	75.270 ± 4.113	< 0.001***

*P < 0.05, **P < 0.01, *** *P* <0.001.

### Metabolomic analysis of human CCs and FF reveals adenosine as a key metabolite associated with the quality of the embryo related to oocyte developmental potential

Metabolomic analysis was conducted on human CCs to investigate the correlation between metabolites and the rate of high-quality embryos. A total of 148 metabolites were measured (Fig. 1C), revealing that 26 of these metabolites exhibited a significant association with the groups based on a high-quality embryo rate of 40% using logistic regression; subsequent linear regression confirmed 15 metabolites (13 with HMDB IDs) remained significantly associated (Table 2). Notably, all metabolites, except for dodecanoic acid, exhibited higher levels in the group with an embryo quality rate of less than 40%. Correlation analysis of the metabolome, along with pathway enrichment analysis, pinpointed purine metabolism and adenosine as key factors linked to embryo quality (Fig. 1D–F). Moreover, changes in adenosine levels in CCs and the corresponding receiver operating characteristic (ROC) curve indicated its biomarker potential (AUC = 0.7465; Fig. 1G, H). Based on these findings, adenosine was selected as the primary focus for further experimental investigations.

**Table 2:** Metabolites related to the rate of high-quality embryos in CCs with consistent results in different regression models.

Metabolite	HMDB ID	OR	*P*^a^	Lower 95% confidence limit^a^	Upper 95% confidence limit^a^	Coefficient^b^	*P*^b^	Lower 95% confidence limit^b^	Upper 95% confidence limit^b^
2-Phenylacetamide	HMDB10715	2.254	0.038	1.048	4.848	−0.153	0.004	−0.255	−0.051
3-Methylhistidine	HMDB00479	29.838	0.004	2.871	310.070	−0.072	0.042	−0.141	−0.003
Adenosine	HMDB00050	2.221	0.048	1.008	4.890	−0.135	0.010	−0.236	−0.034
Benzamide	HMDB04461	3.115	0.010	1.319	7.357	−0.183	0.002	−0.293	−0.073
Benzocaine	HMDB04992	12.438	0.015	1.637	94.517	−0.071	0.042	−0.140	−0.003
Creatinine	HMDB00562	2.082	0.033	1.059	4.091	−0.098	0.038	−0.191	−0.006
Dodecanoic acid	HMDB00638	0.221	0.017	0.064	0.765	0.191	0.019	0.034	0.347
Glycine	HMDB00123	3.460	0.022	1.199	9.979	−0.141	0.007	−0.242	−0.041
L-Carnitine	HMDB00062	4.218	0.015	1.325	13.429	−0.179	0.011	−0.314	−0.044
Levulinic acid	HMDB00720	14.985	0.011	1.845	121.708	−0.102	0.013	−0.181	−0.023
L-Proline	HMDB00162	2.981	0.045	1.026	8.663	−0.194	0.008	−0.334	−0.053
Maleic acid	HMDB00176	7.338	0.025	1.281	42.048	−0.075	0.047	−0.150	−0.001
Sorbitol	HMDB00247	2.348	0.043	1.027	5.369	−0.145	0.005	−0.243	−0.046

^a^Results from logistic regression.

^b^Results from linear regression.

The observation regarding adenosine distribution between CCs and oocytes prompted us to investigate further. Due to ethical constraints, metabolomic analysis was not conducted on human oocytes. To further investigate the distribution of adenosine, we performed targeted metabolomic analysis on FF from 30 couples in two different rates of high-quality embryo groups with matched other factors (Supplementary Table 1). Intriguingly, the relative adenosine level decreased in FF in the high-quality embryo rate < 40% group (Fig. 1I), suggesting that the imbalance of intra- and extracellular adenosine in CCs is a key signature related to oocyte developmental potential in humans.

### Adenosine ameliorates the embryonic development defects of low-quality oocytes

**Fig. 2:**
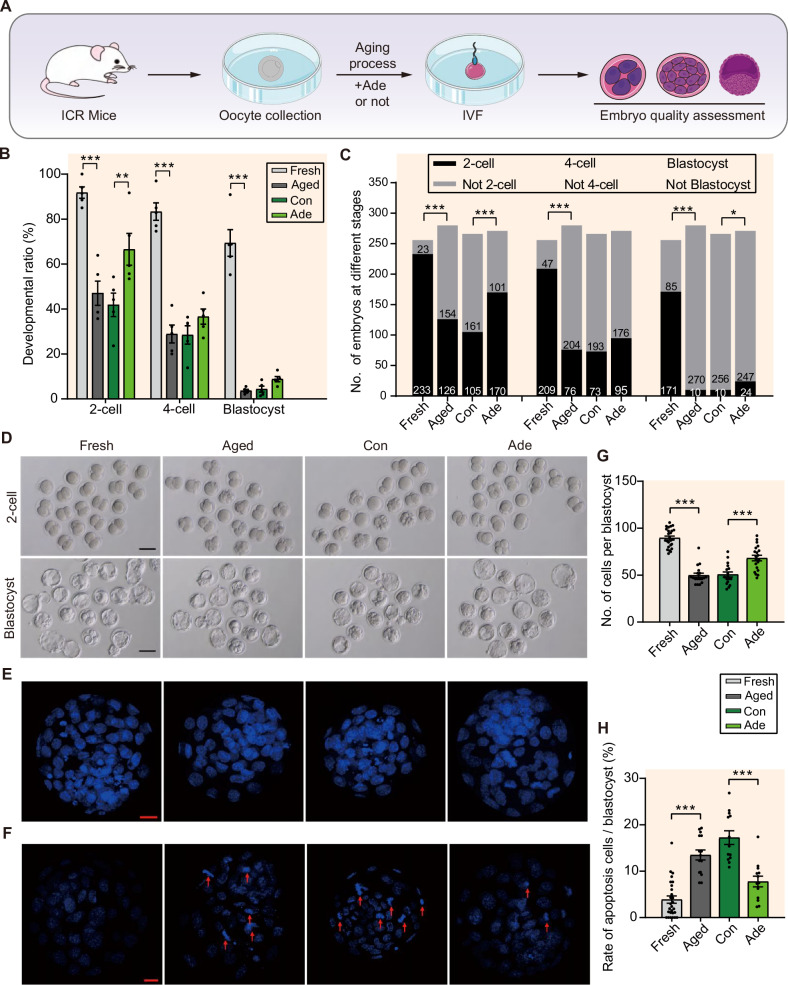
Adenosine restores the developmental potential of embryos derived from low-quality oocytes. **A** A schematic overview of the workflow. **B** The embryonic developmental ratios at different stages were compared among the following groups: Fresh (Fresh control), Aged (aging oocytes), Con (aging oocytes with DMSO), and Ade (aging oocytes with adenosine). The data presented are representative of five independent experiments. **C** The quantification of embryos during the developmental process. **D** Representative images illustrating the developmental process of embryos in various experimental groups. **E** Representative images depicting the total cell count in the blastocyst stage. **F** Representative images depicting apoptotic cells in each blastocyst, highlighted by red arrowheads. **G** The total cell counts in the blastocyst stage. **H** The rate of apoptotic cells within each blastocyst. Statistical analyses were conducted using one-way ANOVA followed by LSD’s multiple comparisons test in (**B**, **G**, **H**) or Fisher’s exact test in (**C**). Scale bar: 20 μm.

To investigate the potential effects of adenosine on oocyte development, we supplemented the medium with adenosine during the in vitro aging of low-quality oocytes (Fig. 2A). After 12 h of in vitro culture, oocytes exhibited significantly lower rates of 2-cell, 4-cell, and blastocyst formation compared to fresh controls. However, adenosine supplementation significantly increased the 2-cell and blastocyst rates (Fig. 2B–D) and improved blastocyst quality by increasing total cell number and reducing apoptotic nuclei (Fig. 2E–H). Taken together, our data show that elevating extracellular adenosine levels can enhance early embryonic development of low-quality oocytes and reduce abnormal embryonic apoptosis, highlighting the importance of normal extracellular adenosine levels for the oocyte to maintain its embryonic developmental potential.

### Adenosine attenuates meiotic defects and oxidative stress in low-quality oocytes

**Fig. 3:**
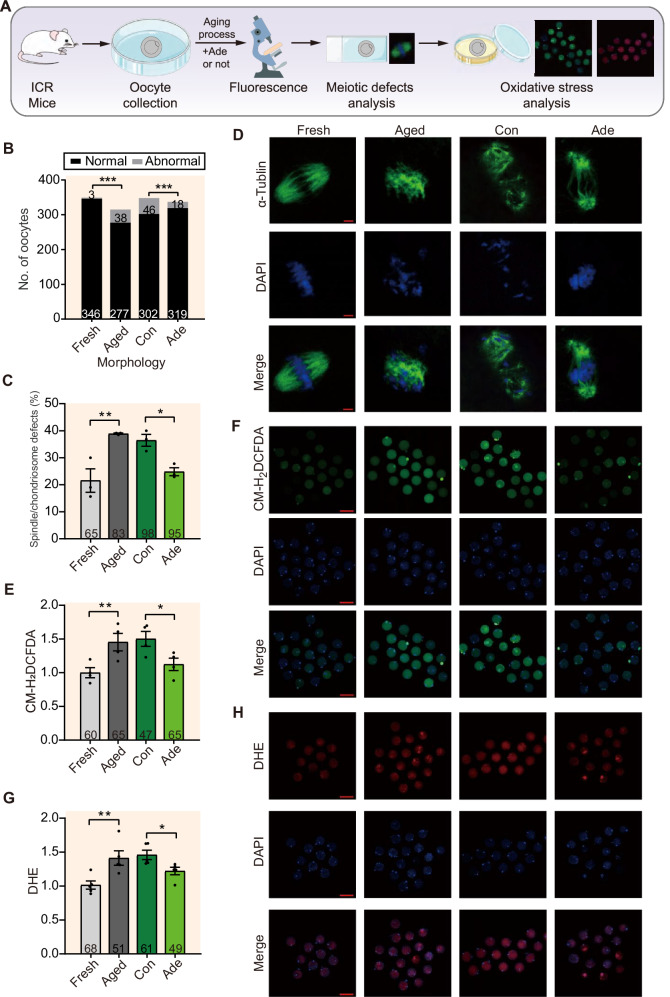
Supplementation with adenosine attenuates meiotic defects and ROS levels in low-quality oocytes. **A** A schematic overview of the workflow. **B** The quantification of oocytes displaying normal and abnormal morphology. **C** The proportions of oocytes exhibiting spindle/chromosome defects. **D** Oocytes were immune-stained with an α-tubulin antibody to visualize the spindles (green) and counterstained with propidium iodide (PI) (blue) to observe chromosomes. Scale bar: 5 μm. **E** Quantification of the relative levels of ROS by CM-H_2_DCFDA fluorescence in oocytes. **F** Representative images depicting CM-H_2_DCFDA fluorescence (green). Scale bar: 100 μm. **G** Quantification of the relative levels of ROS by DHE staining in oocytes. **H** Representative images depicting DHE fluorescence (red). Scale bar: 100 μm. Statistical analyses were conducted using Fisher’s exact test in **B** or one-way ANOVA followed by LSD’s multiple comparisons test in (**C**), (**E**) and (**G**).

To further explore the underlying mechanism, we conducted further experiments to examine the potential of adenosine in improving oocyte quality (Fig. 3A). After 12 h in vitro, abnormal oocytes morphology increased, whereas adenosine effectively reduced the number of abnormal oocytes (Fig. 3B). Previous studies have indicated that the decline of oocyte quality is characterized by a high frequency of spindle defects and chromosomal abnormalities during metaphase [30, 31], adenosine reduced both spindle defects and misalignment (Fig. 3C, D). Additionally, CM-H_2_DCFDA and DHE staining demonstrated lower reactive oxygen species (ROS) in adenosine-treated aged oocytes (Fig. 3E–H), indicating the alleviation of oxidative stress. Collectively, our results suggested that extracellular adenosine supports oocytes developmental potential by preserving morphology, attenuating meiotic defects, and mitigating oxidative stress.

### Adenosine increases in both CCs and oocytes in two independent in vivo models

**Fig. 4:**
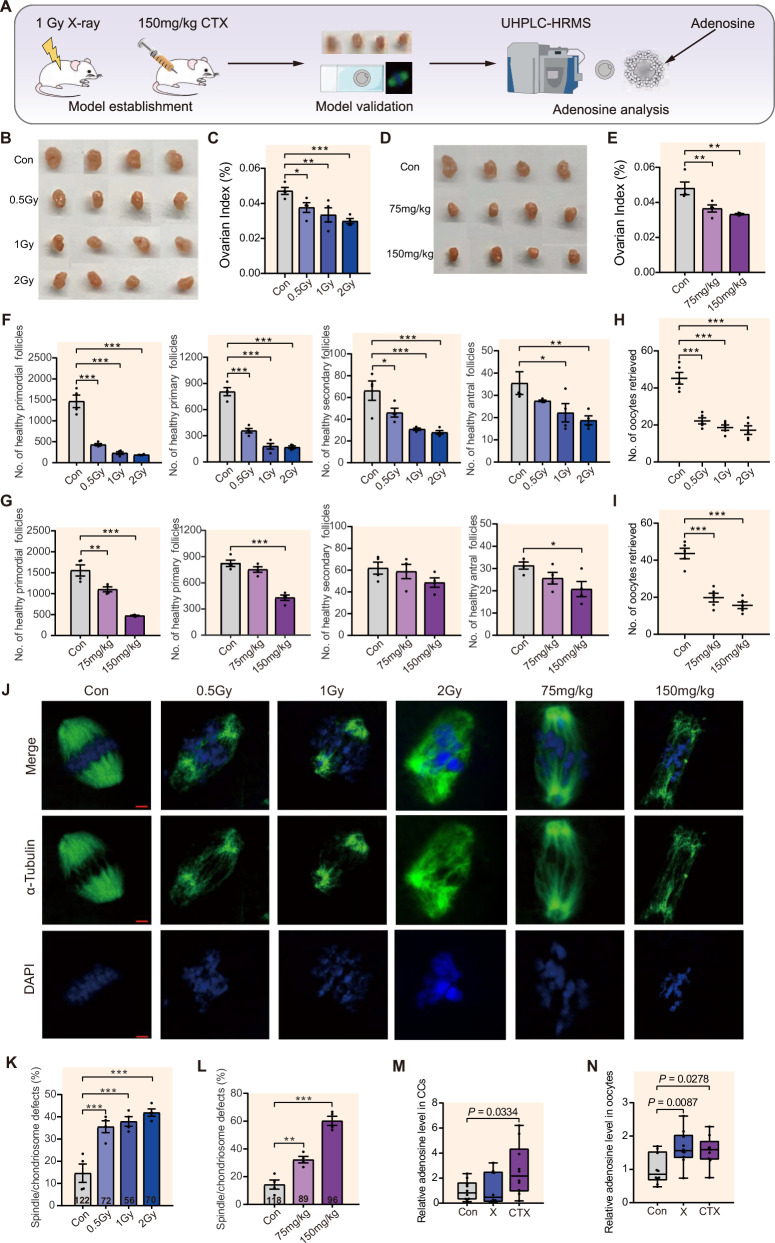
Adenosine levels increase in both CCs and oocytes in X-ray and CTX mouse models of low-quality oocytes. **A** A schematic overview of the workflow to establish a relevant mouse model through whole-body X-ray irradiation or intraperitoneal injection of CTX for adenosine determination. **B** Representative images of mouse ovaries in the X-ray model groups. **C** The ovarian index in the X-ray model groups using the formula: Ovarian index = (wet ovary weight/mouse weight) × 100%. **D** Representative images of mouse ovaries in the CTX model groups. **E** The ovarian index in the CTX model groups. **F** The four stages of healthy follicles in female mice after exposure to 0, 0.5, 1, and 2 Gy X-ray radiation. **G** The four stages of healthy follicles in female mice after intraperitoneally injected with 0, 75, or 150 mg/kg CTX. **H** The number of oocytes retrieved from the X-ray groups. **I** The number of oocytes retrieved from the CTX groups. **J** Oocytes from the control and treatment groups were stained with an α-tubulin antibody to visualize the spindles (green) and counterstained with PI (blue) to observe chromosomes. Scale bar: 5 μm. **K** The proportions of oocytes with spindle/chromosome defects in the X-ray groups. **L** The proportions of oocytes with spindle/chromosome defects in the CTX groups. **M** The relative adenosine levels in CCs of mouse models. **N** The relative adenosine levels in oocytes of mouse models. Statistical analyses were conducted using one-way ANOVA followed by LSD’s multiple comparisons test. The data are presented in interquartile range boxplots in **M**, **N**.

To investigate changes in adenosine levels as oocyte quality and developmental potential decrease in vivo (Fig. 4A), we established mouse models through whole-body X-ray irradiation or intraperitoneal injection of cyclophosphamide (CTX), and determined the optimal dosage for subsequent studies. After one week, we observed a significant decrease in ovarian size and index in both the X-ray and CTX models (Fig. 4B–E; Supplementary Fig. 1A, B and Fig. 2). Furthermore, the number of primordial, primary, secondary, and antral follicles decreased dose-dependently with X-ray exposure (Fig. 4F; Supplementary Fig. 1E) and with CTX administration (significant at 150 mg/kg for primordial, primary, and antral follicles; Fig. 4G; Supplementary Fig. 1F). Additionally, superovulated oocyte yields were reduced (Fig. 4H, I; Supplementary Fig. 1C, D). We also assessed the impact of X-ray and CTX on meiotic structure assembly in oocytes. Similar to the presence of low-quality oocytes with diminished developmental potential in vitro, oocytes from both models exhibited chromosome misalignment and spindle disorganization (Fig. 4J), with increased abnormality rates (Fig. 4K, L). To successfully establish the mouse models in vivo, we selected doses of 1 Gy X-ray and 150 mg/kg CTX based on the observed dose-dependent relationship between treatment and the presence of low-quality oocytes, which exhibited similar characteristics to oocytes with diminished developmental potential in vitro. Subsequent targeted analysis then showed increased adenosine in CCs (Fig. 4M), and elevated adenosine in low-quality oocytes in both models (Fig. 4N). Consistent with animal models, metabolomic data from human populations showed a relatively high adenosine level in CCs and a low adenosine level in FF in relation to low embryo quality (Fig. 1). This indicates an imbalance of intra- and extracellular adenosine not only in CCs but also in oocytes.

### Adenosine transportation gene expression decreases in oocytes and CCs causing the imbalance of intra- and extracellular adenosine

**Fig. 5:**
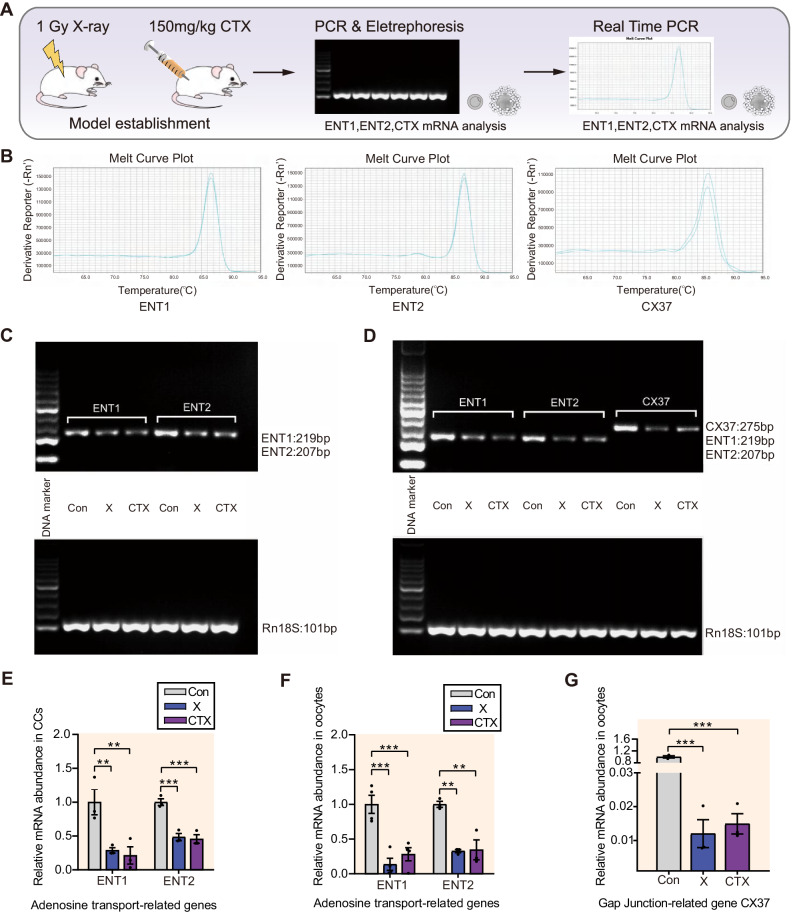
The expression of adenosine transport genes decreases in both X-ray and CTX mouse models of low-quality oocytes. **A** A schematic overview of the workflow for the detection of adenosine transporter genes expression in modeling mouse oocytes and CCs. **B** The melting curves of ENT1, ENT2, and CX37. **C** PCR analysis to assess the mRNA levels of ENT1 and ENT2 in CCs of mouse models. **D** PCR analysis to assess the mRNA levels of ENT1, ENT2, and CX37 in oocytes of mouse models. **E** The relative mRNA abundance of ENTs in CCs. **F** The relative mRNA abundance of ENTs in oocytes. **G** The relative mRNA abundance of CX37 in oocytes. Statistical analyses were conducted using one-way ANOVA followed by LSD’s multiple comparisons test.

We next probed the mechanisms underlying the intra- vs extracellular adenosine imbalance (Fig. 5A). Previous studies have shown that adenosine carrier proteins, including equilibrative nucleoside transporters 1 and 2 (ENT1 and ENT2), are widely distributed on the cell membrane and play a role in adenosine transport. We found that the mRNA levels of ENT1 and ENT2 were significantly reduced in CCs and oocytes following X-ray and CTX treatment (Fig. 5B–F). Furthermore, the relative mRNA abundance of connexin 37 (CX37), a gap junction protein that connects oocytes and CCs, was also markedly decreased after X-ray and CTX treatment (Fig. 5D, G). Therefore, the imbalanced distribution of intra- and extracellular adenosine in low-quality oocytes and their CCs could be attributed to the inhibition of adenosine transport mediated by adenosine carrier proteins in CCs and oocytes, as well as the reduced expression of the CX37 gap junction protein. Consequently, adenosine accumulated in CCs and oocytes, while its levels decreased in FF.

### Adenosine ameliorates embryonic development defects by binding to ARs on the cell membrane

**Fig. 6:**
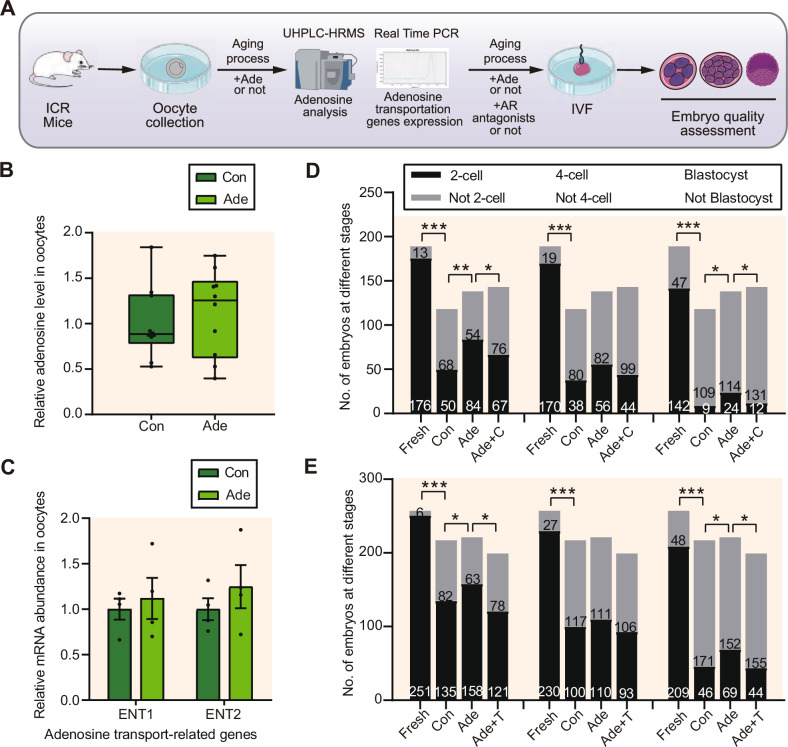
Adenosine binds to its receptors to improve embryonic development. **A** A schematic overview of the workflow for studying the impact of ARs on oocyte IVF. **B** The adenosine content in oocytes following the addition of DMSO or adenosine through an in vitro aging process. **C** The relative mRNA abundance of adenosine transporter genes ENT1 and ENT2 in oocytes following the addition of DMSO or adenosine through an in vitro aging process. **D** Caffeine mitigated the impact of adenosine on embryonic development (Data represented findings from three independent experiments. Ade+C: aging oocytes with adenosine and caffeine). **E** Theophylline mitigated the impact of adenosine on embryonic development (Data represent findings from four independent experiments. Ade+T: aging oocytes with adenosine and theophylline). Statistical analyses were conducted using a two-tailed Student’s *t*-test in **B**, **C** or Fisher’s exact test in **D**, **E**. The data are expressed in (**B**) as interquartile range boxplots.

To investigate the downstream targets of adenosine, we conducted a comprehensive analysis to determine whether adenosine exerts its effects by entering low-quality oocytes or by interacting with adenosine receptors (ARs) on the cell membrane (Fig. 6A). Targeted analysis of adenosine in oocytes revealed no significant change in adenosine levels (Fig. 6B), and transporter mRNA levels in oocytes were unchanged, indicating that adenosine may not exert its effects by direct uptake (Fig. 6C). Notably, ARs located on the oocyte membrane serve as receptors for adenosine and mediate various biological processes. To further examine whether adenosine supplementation functions through its receptors, we supplemented the culture medium with adenosine in combination with caffeine, an AR antagonist [32, 33]. Fertilized oocytes subjected to 12 h with both caffeine and adenosine exhibited significantly lower numbers of 2-cell and blastocyst stage embryos compared to the group treated with adenosine alone (Fig. 6D). To confirm the specificity of these results, we also utilized theophylline, another AR antagonist, and obtained similar findings (Fig. 6E). These results collectively indicate that adenosine enhances developmental capacity primarily via ARs at the oocyte membrane rather than by increasing intracellular adenosine content.

### Adenosine alleviates changes in gene expression involved in oxidative stress and meiotic defects pathways of low-quality oocytes

**Fig. 7:**
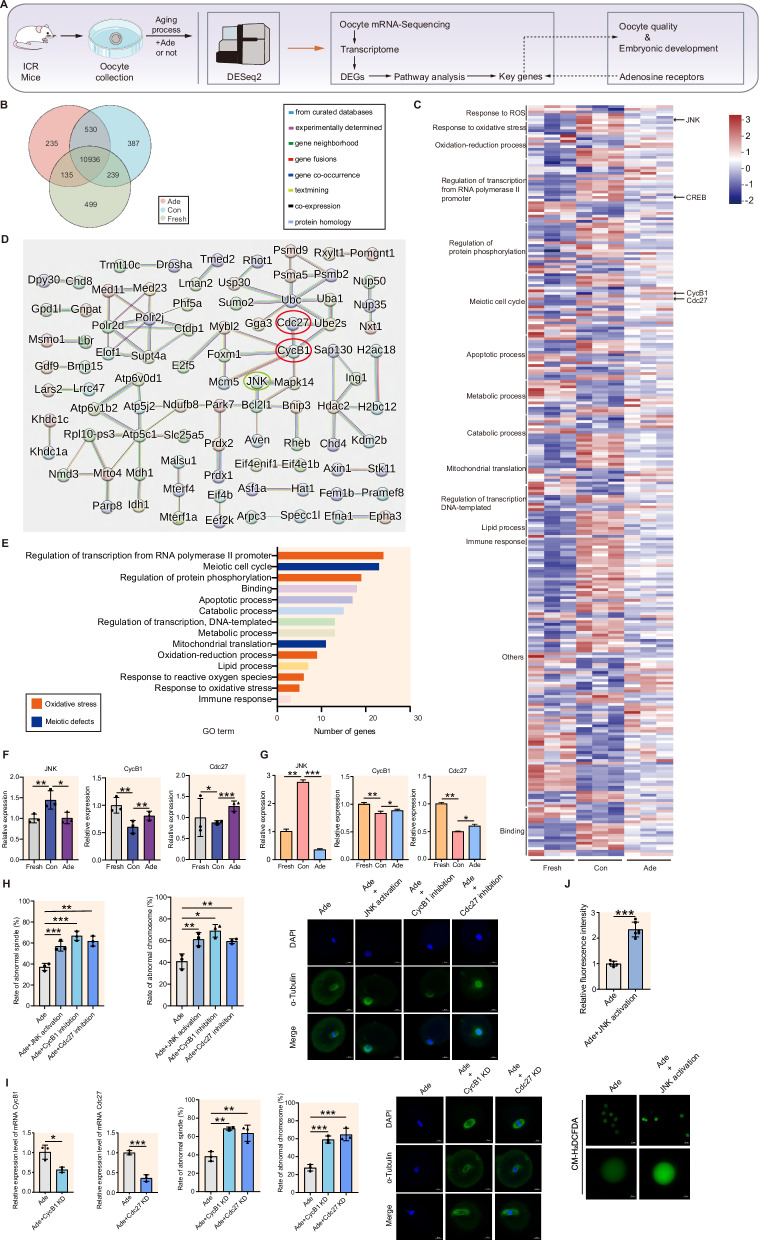
Adenosine mitigates alterations in gene expression related to oxidative stress and meiotic defect pathways in low-quality oocytes. **A** A schematic overview of the transcriptomic study workflow. **B** The study consisted of three groups: Fresh, Con, and Ade groups, and the pie chart displays that these three groups shared a total of 10,936 genes. **C** The heatmap displays 282 genes along with their corresponding pathways. **D** A network diagram depicting the relationships among 282 differentially expressed genes. The thickness and number of lines connecting two genes reflect the closeness and complexity of their relationship. **E** Pathway enrichment analysis revealed that the set of 282 genes was predominantly enriched in pathways related to meiotic defects and oxidative stress. **F** The relative mRNA abundance of key genes, including JNK, CycB1, and Cdc27. **G** The relative mRNA abundance of JNK, CycB1, and Cdc27 in oocytes by RT-PCR. **H** Representative images and quantification showing abnormal spindle formation and chromosome misalignment in oocytes treated with adenosine combined with anisomycin (JNK activator), Ro-3306 (CycB1 inhibitor), or proTAME (Cdc27 inhibitor). Scale bar, 10 μm. **I** Representative images and quantification showing abnormal spindle formation and chromosome misalignment in oocytes treated with adenosine after electroporation with siRNAs targeting CycB1 or Cdc27. Scale bar, 10 μm. **J** Representative images and quantification showing increased ROS levels in oocytes treated with adenosine followed by anisomycin. Scale bar, 10 μm.

Maternal transcriptome remodeling is crucial for meiotic progression and embryonic development, which involves the repression of mRNAs that are active in quiescent oocytes and the activation of mRNAs that are repressed [34]. To investigate the molecular mechanisms by which adenosine influences oocyte function, we conducted a transcriptome analysis employing Smart-seq2 in conjunction with targeted upstream and downstream pathway assessments to identify key genes involved (Fig. 7A). The results, as shown in Supplementary Fig. 3A, B, reveal significant differences in gene expression between the Fresh group and the Con group, indicating that specific genes exhibit altered expression in low-quality oocytes subjected to in vitro aging processes (Supplementary Table 2). Gene Ontology (GO) analysis underscored the relevance of changes related to cell redox homeostasis and microtubule cytoskeleton organization, while Kyoto Encyclopedia of Genes and Genomes (KEGG) analysis highlighted pathways related to signal transduction —a pattern consistent with AR-mediated modulation of oxidative stress and spindle assembly (Supplementary Fig. 3C, D). Subsequently, we focused on the molecular changes related to adenosine intervention.

Three groups, namely Fresh, Con, and Ade groups, had 10,936 genes in common (Fig. 7B). Specifically, adenosine mitigated the dysregulation of 146 up-regulated genes and 136 down-regulated genes in low-quality oocytes (Supplementary Table 3). A detailed heatmap displayed the expression patterns of these 282 genes along with their associated pathways (Fig. 7C). STRING analysis (Fig. 7D) indicated interconnected modules, with enrichment in meiotic process, mitochondrial function, and oxidation processes (Fig. 7E). These pathways are highly relevant to the observed low-quality oocyte feature with increasing ROS levels and meiotic defects. Additionally, the genes were enriched in the regulation of protein phosphorylation, which is related to ARs signal regulation through cyclic adenosine monophosphate (cAMP) -dependent protein kinase A (PKA) pathway. Overall, the transcriptional data provided an overview that supported previous findings, prompting us to further investigate the key specific genes and mechanisms involved.

**Fig. 8:**
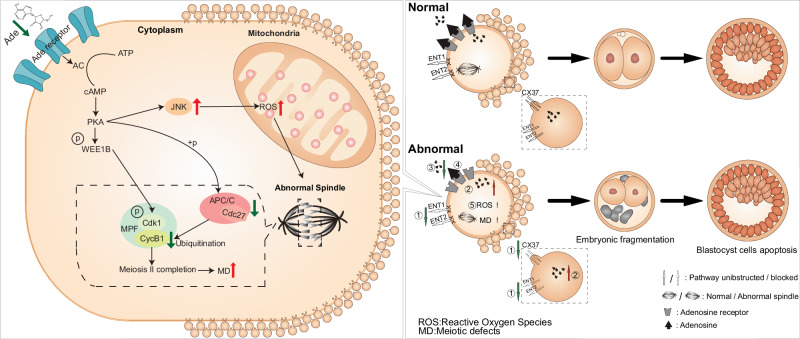
The mechanism underlying the effect of adenosine on oocyte quality and embryonic development. The action of adenosine on oocyte quality and subsequent embryonic development is mediated by several steps. The downregulation of adenosine transporter genes, ENT1 and ENT2, in both oocytes and CCs, as well as the reduced expression of CX37 between oocytes and CCs, leads to the accumulation of adenosine within oocytes and CCs while decreasing its levels in FF. Consequently, this decrease in extracellular adenosine impairs its interaction with ARs, thereby contributing to the generation of ROS through increased JNK expression and meiotic defects through disrupted CycB1 and Cdc27 expression, ultimately compromising oocyte quality and subsequent embryonic development.

To establish the linkage between ARs and meiotic defects/oxidative stress, we employed a comprehensive approach that incorporated biological pathways, such as the KEGG “Oocyte meiosis” pathway. Among the 282 genes analyzed, two downregulated genes, Cyclin B1 (CycB1, gene name: Ccnb1) and Cell Division Cycle 27 (gene name: Cdc27), were found to act as downstream of the cAMP/PKA pathway under adenosine and its receptors regulation, which could eventually trigger meiotic defects. Additionally, these genes were interconnected in the STRING database (Fig. 7D). We also observed an increase in c-Jun N-terminal kinase 1 (JNK, gene name: Mapk8), which is a downstream molecule of the cAMP/PKA pathway under adenosine and its receptors regulation and associated with oxidative stress. The key genes JNK, CycB1, and Cdc27 are highlighted in Fig. 7F. Quantitative RT PCR confirmed these expression changes (Fig. 7G). Furthermore, under the adenosine-rescue condition, we individually perturbed the JNK, CycB1 and Cdc27 to test causality. Activation of JNK (anisomycin), inhibition of CycB1 activity (Ro-3306), or Cdc27 inhibition (proTAME) significantly increased abnormal spindle and chromosome rates compared with adenosine alone (Fig. 7H). Consistently, siRNA-mediated knockdown of CycB1 or Cdc27 similarly blunted the adenosine-mediated rescue and exacerbated meiotic defects (Fig. 7I), with knockdown efficiency confirmed by qRT–PCR (Fig. 7J). In addition, JNK activation in the presence of adenosine markedly elevated ROS levels (Fig. 7J). Collectively, our findings support a model in which adenosine exerts its regulatory effects on the meiotic process through cAMP and its downstream signaling PKA, thereby influencing the expression of CycB1 and Cdc27. In addition, the upregulation of JNK caused by adenosine signaling induces ROS production (Fig. 8).

## Discussion

In this study, we conducted a comprehensive metabolome-wide study encompassing CCs, oocytes, and FF across human and mouse models to identify metabolites associated with high-quality embryos. This approach highlighted adenosine as a crucial metabolite linked to embryo quality. Furthermore, we elucidated the underlying mechanism in which low-quality oocytes exhibit impaired adenosine exchange due to decreased expression of adenosine transporters (ENT1, ENT2) and the gap-junction subunit CX37 in both oocytes and CCs. Consequently, this leads to adenosine accumulation within oocytes and CCs, while depleting the intercellular fluid surrounding these cells. Functionally, exogenous adenosine restored developmental competence by attenuating meiotic defects and oxidative stress through ARs. Additionally, transcriptome profiling implicated JNK, CycB1, and Cdc27 in oocytes as nodes within oxidative stress and meiosis-related pathways influenced by adenosine signaling, thereby providing molecular insights into the mechanisms by which adenosine affects oocyte quality.

Bidirectional interactions among oocytes, CCs, and FF are crucial for oocyte growth, maturation, ovulation, fertilization, and embryonic development [35]. Prior work has highlighted the role of cAMP and the metabolic interdependence between oocytes and CCs, particularly in relation to glucose and lipid metabolism, which are critical for oocyte developmental competence [7, 36]. Our study showed new information regarding changes of intra- and extracellular adenosine concentrations in oocytes, CCs, and FF related to oocyte quality and embryonic development. Adenosine, a ubiquitous signaling molecule and ribonucleotide [37], is phosphorylated by adenosine kinase, metabolized for energy metabolism, and deaminated by adenosine deaminase to generate hypoxanthine, which plays a crucial role in numerous metabolic processes [38]. Notably, adenosine has been shown to delay the resumption of meiosis, allowing for proper chromosome alignment and segregation during oocyte maturation in *Bos taurus* [39]. During this process, adenosine affects mitochondrial function, which is essential for normal oocyte development [39]. Therefore, it is necessary to further study the effects of adenosine on oocyte quality and developmental potential, along with underlying mechanisms.

In our study, we conducted a comprehensive metabolite analysis of FF, CCs, and oocytes, revealing an intriguing finding regarding decreased oocyte quality and the associated intra- and extracellular distribution defect of adenosine. Adenosine accumulation can disrupt important cellular functions and lead to dysregulation; thus, maintaining a balanced adenosine concentration is crucial. Normally, the intra- and extracellular adenosine concentrations can be balanced under normal conditions [40]. However, we found a disruption of this balance in low-quality oocytes and their surrounding CCs and FF. Mechanistically, adenosine crosses cellular membranes via ENTs, which mediate passive, bidirectional flux [41]. ENT1 and ENT2 are pivotal in determining the distribution of adenosine between cells and the extracellular space [42, 43], and reduced expression of these transporters can cause the accumulation of intracellular adenosine [44]. A study has demonstrated that in the murine cardiomyocyte HL-1 cell line, hypoxic conditions result in increased intracellular adenosine levels through ENT1, involving AR-dependent mechanisms [45]. Our study found decreased ENT1 and ENT2 mRNA in low-quality oocytes with limited developmental potential and CCs in two mouse models, indicating the mechanism underlying the imbalance of intra- and extracellular adenosine. Moreover, we observed a dramatic reduction in the relative mRNA abundance of the gap junction-related gene CX37 between oocytes and CCs in low-quality oocytes. Consequently, CCs were unable to effectively transfer small molecular substances like adenosine [46], exacerbating the intra- and extracellular distribution defect of adenosine. These findings provide novel insights into the adenosine imbalance among FF, CCs, and oocytes, highlighting its relevance to embryonic developmental competence.

Beyond its nucleotide role, adenosine acts as an extracellular signal through G-protein-coupled ARs [47]. On the oocyte membrane of *Xenopus*, endogenous ARs modulate GIRK channels via Gi/o [48], and nanomolar adenosine can engage Gi/o and Gs signaling during oocyte maturation. Previous reports have suggested that exogenous adenosine can delay meiotic resumption by binding to its receptors on CCs through a calcium-mediated signaling pathway [49]. Furthermore, adenosine can directly affect mouse oocytes by binding to receptors like the A2 receptor, thereby stimulating adenylate cyclase and generating elevated levels of cAMP, which subsequently arrests meiosis [50]. However, the effects of ARs on oocytes are complex, as their affinity for adenosine varies among different receptor subtypes, and the resultant effects depend on the specific type of AR activated [51]. In our study, we revealed that decreased extracellular adenosine levels may act as regulatory molecules through ARs, leading to a decline in the oocyte quality and embryonic developmental potential. This finding enhances our understanding of the relationship between the adenosine signaling pathway and oocyte-related embryonic developmental competence.

Mammalian antral follicle oocytes, which lack glycolysis and require ATP for meiotic maturation and embryonic development until the blastocyst stage, possess a greater number of mitochondria compared to somatic cells and sperm. This heightened mitochondrial presence makes oocytes particularly susceptible to perturbations that can induce oxidative stress, ultimately compromising meiosis [7]. Oxidative stress refers to an imbalance between pro-oxidant and antioxidant factors in the cellular environment, and it is involved in various physiological and pathological processes [52, 53]. Physiological levels of ROS support gamete function and development [54], excess ROS leads to inflammation, oxidative stress, and detrimental effects on oocyte function, antioxidant mechanisms, ROS homeostasis, and metabolism, as well as lipid peroxidation and mitochondrial dysfunction [55, 56]. Studies have demonstrated that ROS-induced follicular damage results in oocyte apoptosis, spindle abnormalities, DNA fragmentation, and meiotic defects [57, 58]. Combelles et al. have found that the levels of ROS in FF can predict oocyte quality and potential embryonic development [59]. The addition of antioxidants into the in vitro culture system of oocytes has proven to be an effective approach in preventing ROS accumulation [58]. Adenosine possesses antioxidant properties through Nrf2 and Bcl-2-mediated induction of oxidative stress-related proteins [60], although our adenosine recovery experiments did not detect increased intracellular adenosine. Therefore, the recovery effect is attributed to adenosine acting through its receptors. Interestingly, several subtypes of ARs have the potential against oxidative stress, indicating that adenosine possesses antioxidant properties, may be also through its receptors [61, 62]. Therefore, our study combined results from in vitro and two in vivo models to characterize oocytes lacking embryonic development potential. These oocytes exhibited increased embryonic apoptosis and oocyte meiotic arrest due to spindle defects and chromosome misalignment caused by oxidative stress. Remarkably, adenosine was able to alleviate oxidative stress and reverse these adverse changes through its receptor-mediated pathways.

The process of oocyte meiosis and oxidative stress is regulated by gene expression. In female mammals, meiosis occurs over an extended period, with germinal vesicle (GV) oocytes initiating meiotic resumption, expelling the first polar body, and reaching metaphase II only after the preovulatory luteinizing hormone (LH) surge, thereby becoming ready for fertilization. Due to the relatively short lifespan of the mature and fertilizable oocyte within the female reproductive tract, precise regulation of the timing of oocyte meiotic arrest and maturation is essential [63]. As meiotic progression requires temporal regulation of gene expression and analyzing these genes at mRNA levels may allow for elucidating the molecular background [63, 64], we conducted transcriptome analysis to identify the molecules linking adenosine signal and oocyte meiotic defects in low-quality oocytes. When adenosine binds to its receptor, it can modulate the activity of adenylate cyclase, leading to alterations in intracellular cAMP levels [48]. The changes in cAMP levels subsequently influence downstream signaling pathways, including the activation of PKA [65]. PKA balances the activities of Wee1B kinase and Cdc25 phosphatase, and thus regulates the activity of cyclin-dependent kinase 1 (Cdk1) [66]. The CycB1 Cdk1 complex forms the maturation-promoting factor (MPF) complex, which plays a pivotal role in oocytes during their maturation [67]. In the present study, we observed a decreased expression of ARs downstream gene CycB1 in low-quality oocytes, which may lead to impaired completion of meiosis, fertilization, and subsequent embryonic development. The degradation of CycB1 is regulated by the anaphase-promoting complex/cyclosome (APC/C) [68], a ubiquitin ligase that marks CycB1 for proteasomal degradation. This process results in the deactivation of MPF, allowing the oocyte to progress through anaphase II and complete meiosis II [69, 70]. APC/C complex consists of three subcomplexes: a scaffolding sub-complex, a catalytic and substrate recognition sub-complex, and a tetratricopeptide repeat (TPR) arm. The scaffolding subcomplex is composed of APC1, APC4, and APC5; the catalytic sub-complex contains APC2, APC11 (the RING finger protein), and APC10/Doc1; the TPR arm consists of APC3/Cdc27, APC6/Cdc16 and APC8/Cdc23, which provides binding sites for the scaffolding subunit and one of the co-activators (Cdc20 or Cdh1). Activation of PKA leads to the phosphorylation of Cdc20, which restricts APC/C activity, potentially by preventing the interaction of Cdc20 with some of its substrates [71]. Our findings revealed that decreased expression of ARs downstream gene Cdc27 was also observed in low-quality oocytes, potentially disrupting APC/C formation and MPF degradation by targeting CycB1 and subsequently affecting the completion of meiosis, fertilization, and embryonic development. PKA activation can induce the phosphorylation and activation of upstream components in the JNK pathway, thereby contributing to JNK activation [72]. Activation of JNK can stimulate the expression and activity of various ROS-producing enzymes, including NADPH oxidases and mitochondria-associated sources, leading to elevated ROS levels [73]. In our current study, we observed an increase in JNK levels in low-quality oocytes, which can be reversed by adenosine intervention, indicating the role of JNK in adenosine signaling-induced ROS in low-quality oocytes. Consistently, under the adenosine-rescue condition, our functional interventions support JNK, CycB1, and Cdc27 as causal downstream effectors of adenosine signaling (Fig. 7H–J).

In this study, we investigated the potential of adenosine levels in CCs as a predictor for embryo quality. Our findings suggest that CC-derived adenosine may serve as a valuable biomarker for assessing embryo quality in ART. Additionally, optimizing the culture system could further enhance the quality of oocytes and embryos, given the significant influence of the culture environment on their growth and development [74]. During oocyte IVM, melatonin can alleviate high levels of oxidative stress and meiotic defects caused by high fat [75]. Plant-derived quercetin and N-acetyl-L-cysteine can maintain the quality of oocytes, reduce the accumulation of morphological changes and oxidative stress in oocytes caused by aging [76, 77]. In our study, we found that adenosine levels in FF decrease in relation to low-quality embryos and act mainly through ARs on the cell surface. Therefore, optimizing the culture system with adenosine may offer promising avenues for increasing the developmental potential of low-quality oocytes in ART. Future research will focus on elucidating the precise molecular mechanisms underlying the biological functions of adenosine, with the aim of providing novel theoretical insights and practical strategies to advance and innovate ART.

## Methods

### Human experimentation

#### Subjects

The study participants were recruited from the Clinical Center of Reproductive Medicine at the Second Hospital affiliated with Nanjing Medical University. A comprehensive questionnaire was administered to collect relevant patient details. The sample size was estimated based on the high-quality embryo rate using power analysis (two-tailed α = 0.05, power = 80%), with the effect size derived from preliminary experiments. Following a thorough explanation of the study and addressing any inquiries, a total of 43 subjects (approximately 86.0% of the invited individuals) agreed to participate. Written informed consent was signed by each participant. Seven patients were excluded from the study due to a history of polycystic ovary syndrome, endometriosis, ovarian surgery, premature ovarian failure, or chromosomal abnormalities (Supplementary Fig. 4A). These exclusion criteria were pre-established prior to analysis. CCs were obtained from 36 patients undergoing IVF treatment from November 2015 to June 2016, with subsequent follow-up on their IVF outcomes. During the isolation of oocytes from FF for fertilization, a glass pipette was used to excise CCs around approximately 5 ~ 6 oocytes. The collected CCs were washed with phosphate-buffered saline (PBS) and stored at −80°C prior to analysis. FF samples were obtained from 30 patients undergoing IVF treatment from September 2016 to February 2017, following a collection process consistent with that of CCs (Supplementary Fig. 4B). After oocytes removal, FF was collected and subjected to centrifugation at 1000 × *g* for 10 min. The resulting supernatant was carefully collected and stored at −80°C for subsequent analysis. Previous metabolomic studies have demonstrated that a similar sample size within the population can yield valuable insights into metabolic alterations [78, 79].

### The classification of human high-quality embryos

Embryo quality was assessed on day 3 based on the number of blastomeres and fragmentation rate. Grade I embryos have uniform blastomeres and fewer than 5% fragments, Grade II embryos have uniform blastomeres and 5–20% fragments, Grade III embryos have non-uniform blastomeres and 20–50% fragments, and Grade IV embryos have non-uniform blastomeres and more than 50% fragments (Fig. 1B) [80]. High-quality embryos were identified by their grading as Grade I and Grade II. To determine the rate of high-quality embryos, we calculated the number of embryos with Grade I or II ratings, divided by the total number of embryos with normal cleavage, and multiplied the result by 100%.

### Metabolomic analysis

The metabolomic analysis was conducted following previously reported methods [81]. Briefly, a methanol/water mixture was added to the CCs, followed by homogenization in an ice-water bath. Subsequently, ultrasonication was used to disrupt all the cells for 1 min (power: 60%, pulses: 3/3). The samples were then centrifuged at 15,000 × *g* for 15 min at 4˚C, and the supernatant was evaporated to dryness. For LC-HRMS analysis, a 10 μl aliquot of the reconstituted sample was injected. The LC-HRMS analysis was performed using a UPLC Ultimate 3000 system (Dionex) coupled with a Q-Exactive mass spectrometer (Thermo Fisher Scientific, Bremen, Germany). The UPLC analysis utilized a multistep gradient with mobile phase A containing 0.1% formic acid in ultrapure water and mobile phase B consisting of acetonitrile acidified with 0.1% formic acid, flowing at a rate of 0.4 ml/min over a 15 min run time. The mass spectrometer operated at a resolution of 70,000 using full-scan acquisition ranging from 70 to 1050 m/z. To minimize potential bias from the injection order, all samples were analyzed randomly. Metabolite identification was based on accurate mass and retention time compared with commercial metabolite standards.

### Targeted adenosine analysis

The adenosine concentrations in FF and oocytes were quantified using LC-HRMS following the same experimental conditions as described above for metabolomic analysis. The mass spectrometer and chromatograph parameters remained consistent.

### Animal experimentation

#### Animals

The experimental mice for this study were obtained from the Animal Core Facility of Nanjing Medical University (Nanjing, China). Prior to the commencement of the experiments, the animals underwent a 1-week acclimation period within the facility. Throughout the study, the mice were housed in a controlled environment with constant temperature (23 ± 1°C) and humidity (53 ± 2%), and maintained on a 12 h light/dark cycle with ad libitum access to food and water. Sample size was estimated based on preliminary experiments and published data, using oocyte quality and embryo development as primary indicators. A sample size of five mice per group was determined to provide adequate statistical power. Mice were randomly assigned to experimental groups.

### In vitro oocyte collection and culture

To obtain MII oocytes, 8-week-old female ICR mice were superovulated through intraperitoneal injection of 5 IU of pregnant mare serum gonadotropin (PMSG) (Ningbo Sansheng, 110044564). After a 48-h interval, 5 IU human chorionic gonadotropin (hCG) (Ningbo Sansheng, 110041282) was injected intraperitoneally. The super-ovulated mice were euthanized 14 h after hCG injection. Oviductal ampullae were carefully torn in M2 medium (Sigma-Aldrich, M7167) to release cumulus-oocyte complexes [82], and CCs were removed by a 5-min incubation in hyaluronidase (Sigma-Aldrich, H1115000). Subsequently, the oocytes were washed three times in M2 medium to ensure complete removal of the CCs. Following the washing steps, cumulus-free oocytes were cultured in M2 medium under a 5% CO_2_ atmosphere at 37 °C.

### Adenosine treatment, AR antagonist, and key gene intervention

In the adenosine treatment protocol, a concentration of 10 μM adenosine (Sigma-Aldrich, A9251, purity ≥ 99%), dissolved in dimethyl sulfoxide (DMSO, Sigma-Aldrich, D8418), was supplemented to the culture medium [83]. The oocytes in the solvent control group were exposed to an equivalent volume of DMSO. To induce aging, fresh MII denuded oocytes were cultured at a density of approximately 40 oocytes per 100 μl drop in M2 medium. The control group received M2 medium with DMSO (solvent control), while the experimental group was treated with M2 medium supplemented with 10 μM adenosine, all under mineral oil (Sigma-Aldrich, M8410) for a duration of 12 h. Adenosine was initially dissolved in DMSO, and subsequently diluted to a final concentration of 10 μM with M2 medium. Additionally, the AR antagonist group was supplemented with either 10 μM caffeine (Sigma-Aldrich, 205548, purity ≥ 99%) or theophylline (Sigma-Aldrich, T1633, purity ≥ 99%), in accordance with the treatment provided to the experimental group. The key gene reversal group was supplemented with individual treatment with 100 nM anisomycin (MedChemExpress, HY-18982, purity 99.82%) [84], 500 nM Ro-3306 (MedChemExpress, HY-12529, purity 99.57%) [85] or 5 nM proTAME (MedChemExpress, HY-124955, purity 97.33%) [86] to activate JNK and inhibit CycB1 and Cdc27, in accordance with the treatment provided to the experimental group. The key gene knockdown group was also supplemented, in accordance with the treatment provided to the experimental group. Denuded oocytes were randomized to siCycB1, siCdc27, or non-targeting control siRNA groups (~30–50 oocytes/group/replicate; two biological replicates). Oocytes were mixed with siRNA (150 nM, IBSBio, Shanghai, China) in electroporation buffer and electroporated in a Lonza 4D-Nucleofector 16-well Nucleocuvette (10 μL/well) using a mild short-pulse program [87, 88].

### In vitro fertilization

Sperm samples were retrieved from the cauda epididymides of male ICR mice and capacitated in fertilization medium (Human Tubal Fluid containing 1% BSA) for 1.5 h at 37°C and 5% CO_2_ under mineral oil. Prior to completing the capacitation process, oocytes that had been aged for 12 h were washed and transferred to the fertilization medium. Additionally, freshly released oocytes were also fertilized and used as a control. Approximately 4-5 μl of capacitated sperm were added to the fertilization dishes of both the control and experimental groups. Following a fertilization period of 4–5 h, the oocytes were washed three times using human tubal fluid (HTF, Irvine Scientific, 90125) and incubated overnight until they reached the 2-cell stage. On the morning of day 3, the 2-cell embryos progressed to the 4-cell stage and were then transferred to pre-equilibrated KSOM medium (Millipore, MR-020P-D). On days 4 and 5, the 4-cell embryos had reached the morula and blastocyst stages, respectively.

### Evaluation of blastocyst quality

To evaluate the quality of blastocysts, they were fixed in a solution consisting of 4% paraformaldehyde in PBS for 30 minutes at room temperature. Subsequently, the blastocysts were washed three times for 5 min each, using PBS containing 0.1% Tween-20 and 0.01% Triton X-100. The nuclear status of cells present within the blastocysts was then visualized by staining with Hoechst 33342 stain (Beyotime, C1022), or cell apoptosis-Hoechst (Beyotime, C0003) at room temperature, for a period of 5 min. After a brief washing step, the blastocysts were mounted on glass slides in a drop of polyvinylpyrrolidone anti-fade medium (Beyotime, P0123). Finally, the blastocysts were examined using a Zeiss LSM 700 laser scanning confocal microscope.

### Spindle and chromosome alignment detection

For spindle staining, MII oocytes were first fixed with 4% paraformaldehyde in PBS at room temperature for 30 min. Subsequently, the oocytes were transferred to membrane permeabilization using a solution of 0.5% Triton X-100 in PBS at 37 °C for 30 min. Following this, the oocytes were blocked in a solution composed of PBS with 1% BSA, 0.1% Tween-20, and 0.01% Triton X-100 at room temperature for 1 h. The oocytes were then incubated overnight at 4 °C with anti-α-Tubulin-FITC antibody (Sigma-Aldrich, F2168) at a dilution of 1:500 in blocking buffer. After incubation, the oocytes were washed three times for 5 min each, using PBS containing 0.1% Tween-20 and 0.01% Tritonx-100. The nuclear status of the oocytes was visualized by staining with Hoechst 33342 (Beyotime, C1022) at room temperature for 5 min. Following a brief washing step, the oocytes were mounted on glass slides in a drop of polyvinylpyrrolidone anti-fade medium (Beyotime, P0123). Finally, the oocytes were examined using a Zeiss LSM 700 laser scanning confocal microscope.

### Measurement of the intracellular ROS level

To assess the level of intracellular ROS, we utilized 5-(and-6)-chloromethyl-2′,7′-dichlorodihydrofluorescein diacetate (CM-H_2_DCFDA, Invitrogen) and dihydroethidium (DHE, Vigorous Biotechnology). MII oocytes from distinct treatment groups were subjected to a 30–40 min incubation at 37 °C in M2 medium containing 5 μM CM-H_2_DCFDA or 25 μM DHE. Following this, the oocytes underwent these washes, and their nuclei were stained with Hoechst 33342 at room temperature for 5 min. Subsequently, after a brief washing step, the oocytes from both the control and experimental groups were positioned within the same dishes, and imaging was conducted using a Zeiss LSM 700 confocal microscope with consistent scanning parameters. ImageJ software (NIH) was employed to evaluate the fluorescence intensity of the oocytes. The fluorescence intensity of every oocyte was quantified, and the average level across all measurements was compared between the control and experimental groups.

### In vivo treatment and ovarian histomorphometric analysis

A model of diminished oocyte quality in female mice was developed through exposure to X-ray radiation or treatment with CTX, following a documented protocol [89, 90]. Seven-week-old female ICR mice were subjected to X-ray doses of 0, 0.5, 1, or 2 Gy, or received intraperitoneal injections of CTX at doses of 0, 75, or 150 mg/kg. Subsequently, the mice were maintained on a standard diet for 1 week before being humanely euthanized.

After euthanasia of the mice, ovarian tissue was extracted and fixed in 4% paraformaldehyde in PBS for 24 h, followed by preservation in 70% ethanol until embedding in paraffin. The embedded ovarian tissue samples were serially sectioned at a thickness of 5 μm through the entire tissue. Subsequently, the sections were stained with hematoxylin and eosin. An observer, blinded to the treatment groups, quantified the ovarian follicles using light microscopy. The follicles were categorized as primordial, primary, secondary, or antral. Primordial, primary, and secondary follicles were counted in every fifth serial section. To prevent duplication, primordial and small primary follicles were only tallied when the oocyte nucleus was clearly visible, while larger primary and secondary follicles were only tallied when the oocyte nucleolus was clearly visible. Antral follicles were tracked through every section, ensuring each antral follicle was counted only once.

### Extraction of total RNA, reverse transcription into cDNA, and quantitative RT-PCR

To extract total RNA from oocytes and CCs, a RNeasy Plus Micro kit (QIAGEN GmbH, 74034) was utilized. The extracted RNA was reverse-transcribed into complementary DNA (cDNA) using the Superscript III reverse transcriptase enzyme (Takara Bio Inc., Otsu, Shiga, Japan). A housekeeping gene, glyceraldehyde-3-phosphate dehydrogenase (GAPDH), was employed as an internal control. The primer sequences used in this study are listed in Supplementary Table 4, and the corresponding melting curves are presented in Fig. 5B. Gene expression was detected through the use of qPCR SYBR Green Master Mix (Vazyme Biotech Co., Q111-02). Data were analyzed using the 2^-ΔΔCT^ method, and mRNA abundance was evaluated relative to an internal control.

### Agarose gel electrophoresis

Agarose (Biosharp, Hefei, China, #BS081-100g) weighing 1.6 g was dissolved in 80 ml of 1× TAE buffer (Biosharp, Hefei, China, #BL533A) and heated until fully dissolved. The solution was allowed to cool to approximately 50 °C before 10 μl of Goldview nucleic acid dye (Biosharp, Hefei, China, #BS357A) was added and mixed thoroughly. The prepared agarose solution was then poured gently into the electrophoresis tank along the walls, with the comb inserted immediately to create wells. After gel solidification, the comb was carefully removed, and the gel plate was placed in the electrophoresis tank. Electrophoresis solution was added, followed by the appropriate volume of PCR product and loading buffer into the sample wells, while marker wells were designated as control wells. Adding electrophoresis solution, adding appropriate volume of PCR products, corresponding primers (Supplementary Table 4), and sample loading buffer in the sample hole, control hole is appropriate volume of PCR products, Rn18S, and sample loading buffer. Gel electrophoresis was performed using a gel imaging system (BIO-RAD, USA) under the following conditions: 110 V for 40 min, with subsequent visualization of outcomes.

### Transcriptomic analysis

Oocytes from fresh, aged, and adenosine-treated groups were collected for Smart-seq2 transcriptome assay. Each sample contained 80-100 oocytes, which were lysed in a buffer comprising lysis buffer and RNase inhibitor. Total RNA was extracted using TRIzol® Reagent (Invitrogen) and treated with DNase I (TaKaRa) to remove genomic DNA. Following RNA quality assessment and quantification, transcriptome libraries were generated using the Clontech-SMART-Seq™ v4 Ultra™ Low Input RNA Kit with 10 ng of total RNA, subsequently sequenced using the Illumina NovaSeq 6000 sequencer (2×150 bp read length) to produce paired-end RNA-seq libraries. The expression level of each gene was quantified using the transcripts per million reads (TPM) method, and differential expression analysis was performed using DESeq2, with DEGs having a *P*-adjust < 0.05 with a Benjamini & Hochberg (BH) method considered as significantly differentially expressed genes. Furthermore, functional enrichment analyses, including GO (Gene Ontology, http://www.geneontology.org) and KEGG (Kyoto Encyclopedia of Genes and Genomes, http://www.genome.jp/kegg/), were performed to identify significantly enriched GO terms and metabolic pathways compared with the whole-transcriptome background. The genes associated with oocyte aging and adenosine action were intersected to identify targets influenced by adenosine in mitigating oocyte aging. A Venn diagram was constructed using the bioinformatics platform jvenn (http://jvenn.toulouse.inra.fr/app/index.html), while the protein-protein interaction (PPI) network of common target genes was obtained from the STRING database (https://string-db.org/), limited to *Mus musculus* and requiring a combined score > 0.7. The Cytoscape 3.8.2 software was used to visualize and construct the PPI network. To identify key genes involved in the processes studied, we used a comprehensive approach that incorporated biological pathways, specifically analyzing the KEGG “Oocyte meiosis” pathway to determine genes downstream of the AR/cAMP/PKA pathway and upstream of meiotic defects and oxidative stress.

### Statistical analysis

Statistical analysts were blinded to group allocation during data analysis for both the clinical cohort and animal experiments. Linear and logistic regression models were used to explore the relationship between metabolomic data in human CCs and the rate of high-quality embryos. In the linear regression model, the rate of high-quality embryos was treated as a continuous variable, while in the logistic regression model, it was treated as a binary variable. In the logistic regression analysis, two groups were formed based on a cutoff rate of 40% for high-quality embryos, resulting in matched groups with a 2:1 ratio for comparison. The classification was informed by data from normal control groups from other reproductive centers [28, 29]. Statistical differences between the two groups were evaluated using a two-tailed Student’s t-test, while comparisons among three or more groups were assessed using ANOVA followed by Dunnett’s test. The constituent ratios between groups were compared using Fisher’s exact test. Levene’s test was used to assess the homogeneity of variances prior to parametric tests. Normality was assessed using the Shapiro-Wilk test prior to parametric analyses. The dose-effect relationship between X-ray and CTX treatment dosages and oocyte quality indices was examined through Spearman correlation analysis. Statistical analyses were performed using Stata statistical software (Version 9.2, Stata Corp, LP, Lakeway Drive, College Station, TX, USA) and GraphPad Prism (GraphPad Software). The results are presented as mean ± s.e.m. (standard error of the mean), and statistical significance was defined as *P* < 0.05 (* *P* < 0.05, ** *P* < 0.01, *** *P* < 0.001).

## Supplementary information

Supplementary Fig, 1

Supplementary Fig. 2

Supplementary Fig. 3

Supplementary Fig. 4

Supplementary Fig. legends

Supplementary Table 1

Supplementary Table 2

Supplementary Table 3

Supplementary Table 4

## Data Availability

Source data, including all metabolomic data, are provided as a Source Data file. The Smart-seq2 datasets generated during the current study are available in the Sequence Read Archive, accession code PRJNA1127060. All other unprocessed data supporting the findings of this study are available from the corresponding authors upon reasonable request.
