# Supplementary material for: Metabolomic profiling delineates the role of adenosine in oocyte quality and embryonic development
Source: Cell Death Dis. 2026 Jun 9;17(1):724. doi: 10.1038/s41419-026-08886-9 (PMC13473481; doi:10.1038/s41419-026-08886-9)
Supplement: Supplementary file 1 [file 41419_2026_8886_MOESM1_ESM.pdf]

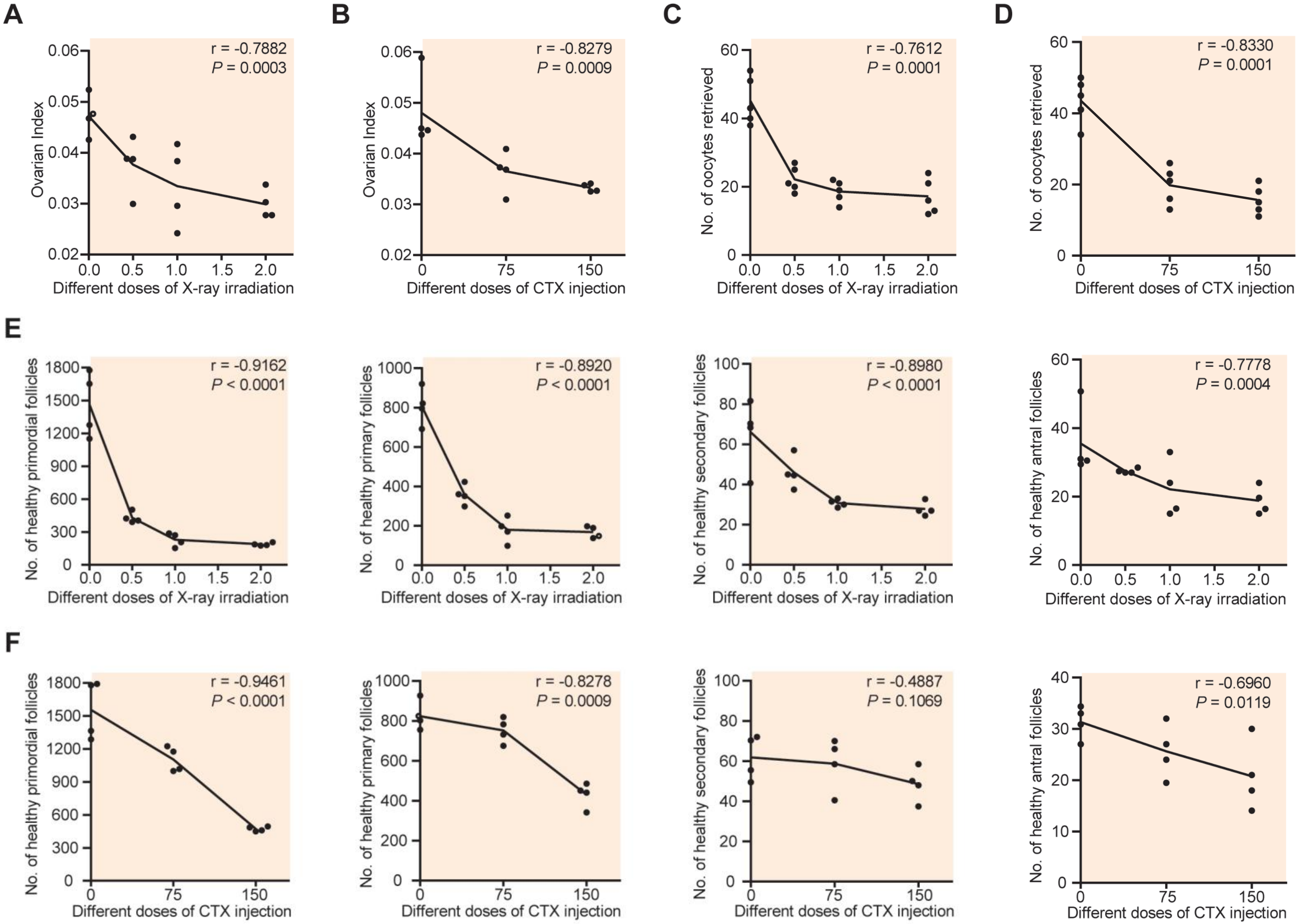

A
r = -0.7882
P = 0.0003
Ovarian Index
Different doses of X-ray irradiation
B
r = -0.8279
P = 0.0009
Ovarian Index
Different doses of CTX injection
C
r = -0.7612
P = 0.0001
No. of oocytes retrieved
Different doses of X-ray irradiation
D
r = -0.8330
P = 0.0001
No. of oocytes retrieved
Different doses of CTX injection
E
r = -0.9162
P < 0.0001
No. of healthy primordial follicles
Different doses of X-ray irradiation
r = -0.8920
P < 0.0001
No. of healthy primary follicles
Different doses of X-ray irradiation
r = -0.8980
P < 0.0001
No. of healthy secondary follicles
Different doses of X-ray irradiation
r = -0.7778
P = 0.0004
No. of healthy antral follicles
Different doses of X-ray irradiation
F
r = -0.9461
P < 0.0001
No. of healthy primordial follicles
Different doses of CTX injection
r = -0.8278
P = 0.0009
No. of healthy primary follicles
Different doses of CTX injection
r = -0.4887
P = 0.1069
No. of healthy secondary follicles
Different doses of CTX injection
r = -0.6960
P = 0.0119
No. of healthy antral follicles
Different doses of CTX injection
