# Supplementary material for: Metabolomic profiling delineates the role of adenosine in oocyte quality and embryonic development
Source: Cell Death Dis. 2026 Jun 9;17(1):724. doi: 10.1038/s41419-026-08886-9 (PMC13473481; doi:10.1038/s41419-026-08886-9)
Supplement: Supplementary file 2 [file 41419_2026_8886_MOESM2_ESM.pdf]

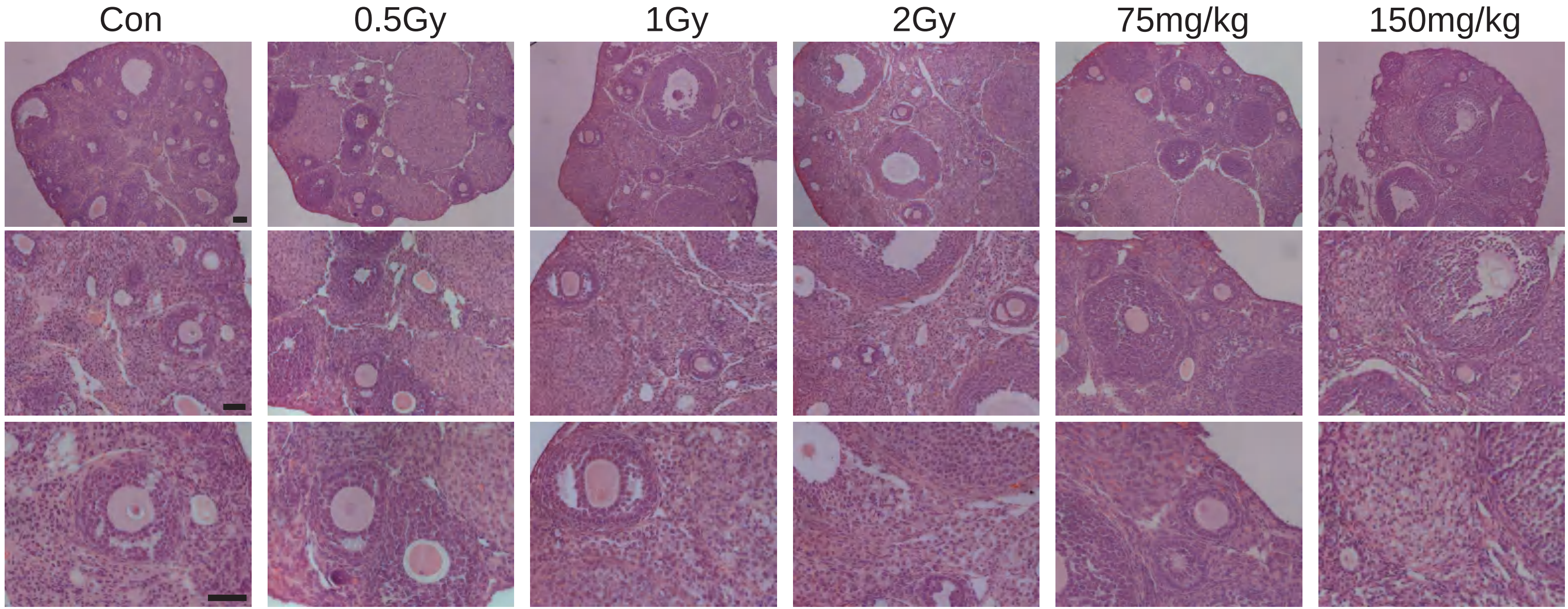
Con
0.5Gy
1Gy
2Gy
75mg/kg
150mg/kg
