# Supplementary material for: Metabolomic profiling delineates the role of adenosine in oocyte quality and embryonic development
Source: Cell Death Dis. 2026 Jun 9;17(1):724. doi: 10.1038/s41419-026-08886-9 (PMC13473481; doi:10.1038/s41419-026-08886-9)
Supplement: Supplementary file 3 [file 41419_2026_8886_MOESM3_ESM.pdf]

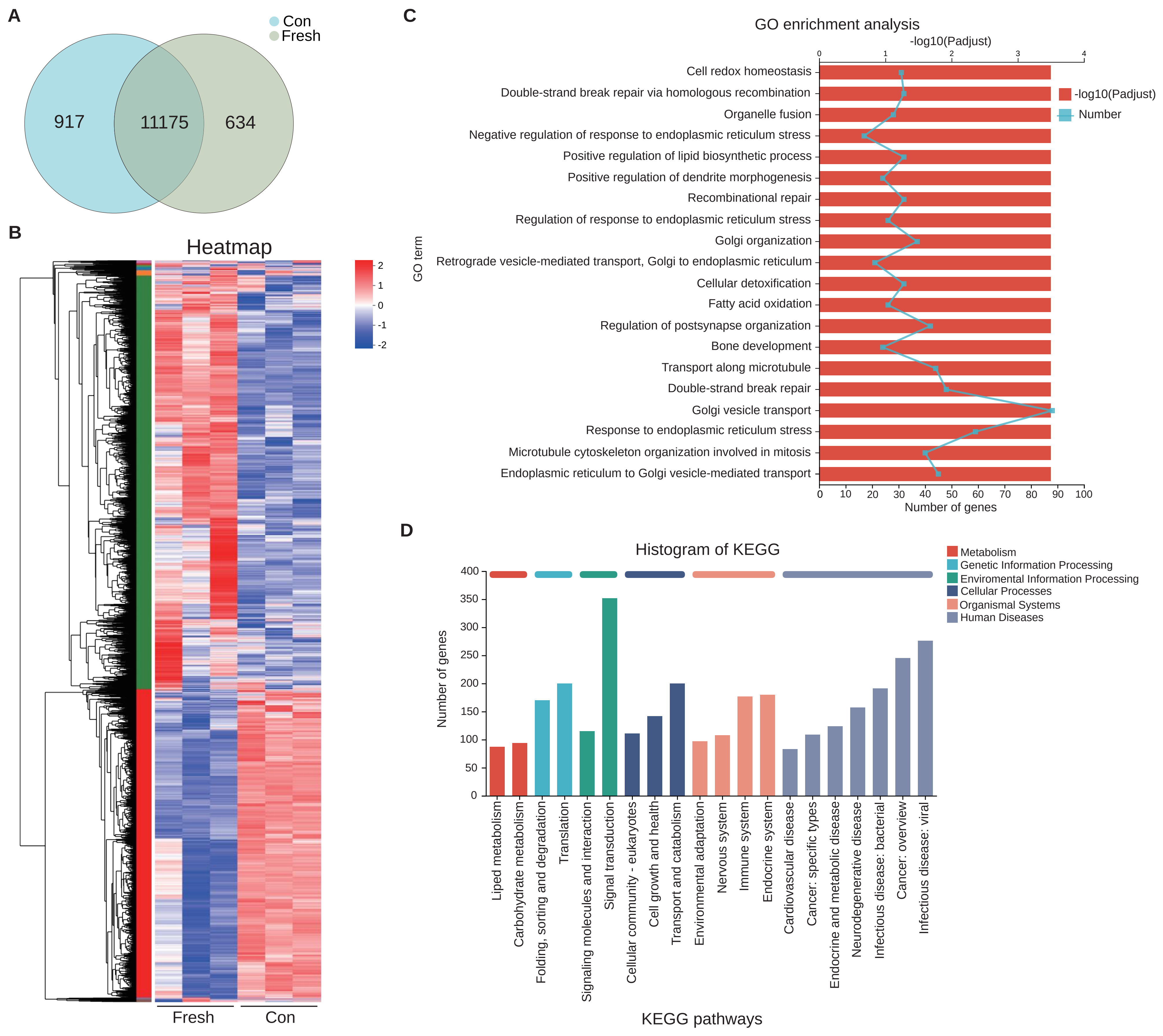
A
Con
Fresh
917
11175
634
B
Heatmap
2
1
0
-1
-2
Fresh
Con
C
GO enrichment analysis
-log10(Padjust)
Cell redox homeostasis
Double-strand break repair via homologous recombination
Organelle fusion
Negative regulation of response to endoplasmic reticulum stress
Positive regulation of lipid biosynthetic process
Positive regulation of dendrite morphogenesis
Recombinational repair
Regulation of response to endoplasmic reticulum stress
Golgi organization
Retrograde vesicle-mediated transport, Golgi to endoplasmic reticulum
Cellular detoxification
Fatty acid oxidation
Regulation of postsynapse organization
Bone development
Transport along microtubule
Double-strand break repair
Golgi vesicle transport
Response to endoplasmic reticulum stress
Microtubule cytoskeleton organization involved in mitosis
Endoplasmic reticulum to Golgi vesicle-mediated transport
GO term
Number
Number of genes
D
Histogram of KEGG
Metabolism
Genetic Information Processing
Enviromental Information Processing
Cellular Processes
Organismal Systems
Human Diseases
Number of genes
Liped metabolism
Carbohydrate metabolism
Folding, sorting and degradation
Translation
Signaling molecules and interaction
Signal transduction
Cellular community - eukaryotes
Cell growth and health
Transport and catabolism
Environmental adaptation
Nervous system
Immune system
Endocrine system
Cardiovascular disease
Cancer: specific types
Endocrine and metabolic disease
Neurodegenerative disease
Infectious disease: bacterial
Cancer: overview
Infectious disease: viral
KEGG pathways
