# Supplementary material for: Metabolomic profiling delineates the role of adenosine in oocyte quality and embryonic development
Source: Cell Death Dis. 2026 Jun 9;17(1):724. doi: 10.1038/s41419-026-08886-9 (PMC13473481; doi:10.1038/s41419-026-08886-9)
Supplement: Supplementary file 4 [file 41419_2026_8886_MOESM4_ESM.pdf]

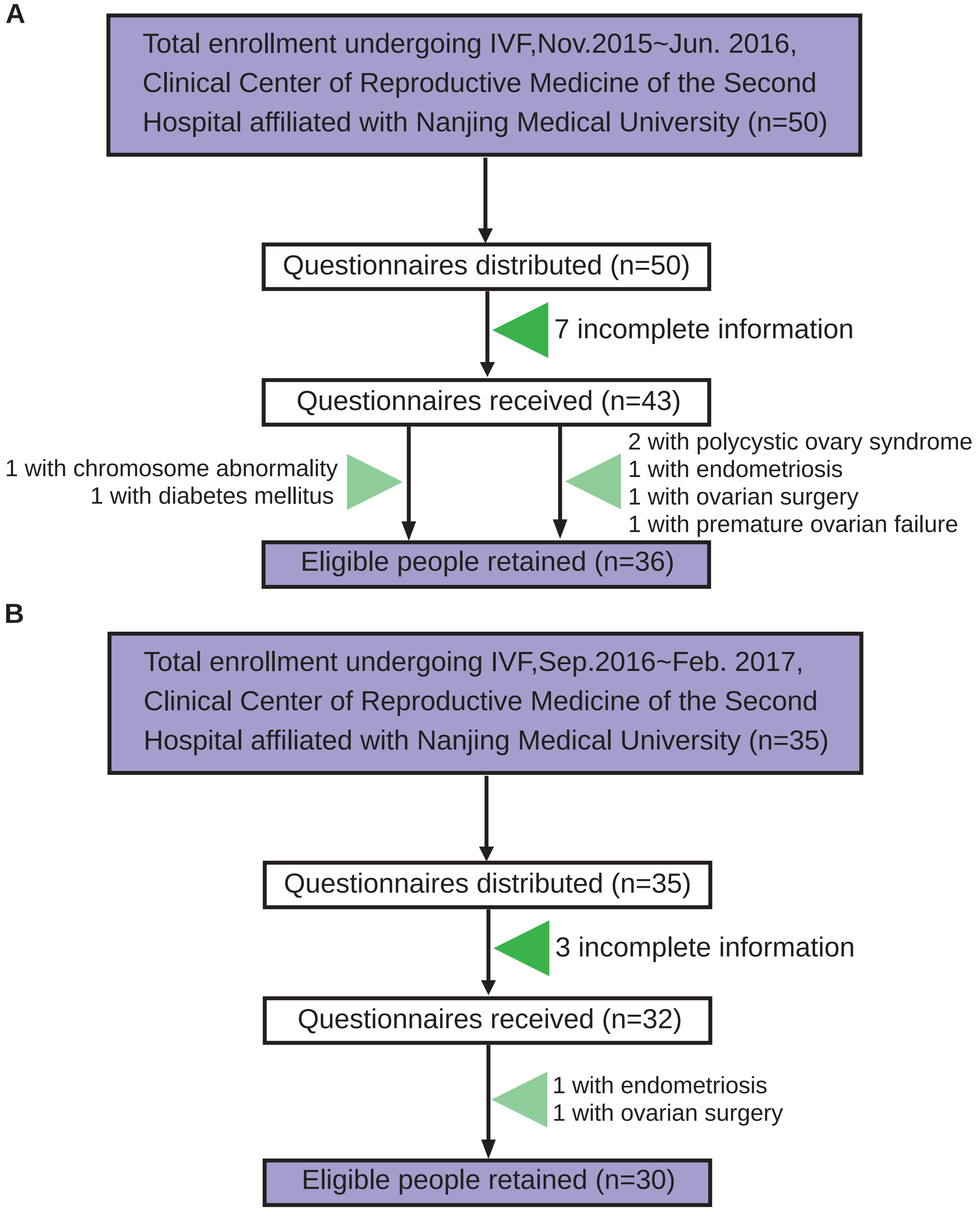
A
Total enrollment undergoing IVF,Nov.2015~Jun. 2016, Clinical Center of Reproductive Medicine of the Second Hospital affiliated with Nanjing Medical University (n=50)
Questionnaires distributed (n=50)
7 incomplete information
Questionnaires received (n=43)
1 with chromosome abnormality
1 with diabetes mellitus
2 with polycystic ovary syndrome
1 with endometriosis
1 with ovarian surgery
1 with premature ovarian failure
Eligible people retained (n=36)
B
Total enrollment undergoing IVF,Sep.2016~Feb. 2017, Clinical Center of Reproductive Medicine of the Second Hospital affiliated with Nanjing Medical University (n=35)
Questionnaires distributed (n=35)
3 incomplete information
Questionnaires received (n=32)
1 with endometriosis
1 with ovarian surgery
Eligible people retained (n=30)
