# Supplementary material for: Metabolomic profiling delineates the role of adenosine in oocyte quality and embryonic development
Source: Cell Death Dis. 2026 Jun 9;17(1):724. doi: 10.1038/s41419-026-08886-9 (PMC13473481; doi:10.1038/s41419-026-08886-9)
Supplement: Supplementary file 5 [file 41419_2026_8886_MOESM5_ESM.docx]

**Supplementary Fig. 1 X-ray and CTX induce depletion of ovarian follicles. A** Ovary index in X-ray model group (wet ovary weight/mouse weight ×100%). **B** Ovary index in CTX model group. **C** The number of oocytes retrieved in X-ray model group. **D** The number of oocytes retrieved in CTX model group. **E** The number of healthy follicles at each stage, including primordial, primary, secondary and antral follicles in X-ray model group. **F** The number of healthy follicles at each stage, including primordial, primary, secondary and antral follicles in CTX model group. **P* < 0.05, ***P* < 0.01, ****P* < 0.001, compared with control.

**Supplementary Fig. 2 General morphology of mouse ovaries in X-ray and CTX model groups.**

**Supplementary Fig. 3 Effects of aging on gene expression levels in mouse oocytes. A** Relationship between the number of genes detected in Fresh and Con groups. **B** The heatmap of gene clustering in Fresh and Con groups. **C** GO enrichment pathway map for differentially expressed genes in Fresh and Con groups. **D** Histogram of KEGG of differentially expressed genes in Fresh and Con groups.

**Supplementary Fig. 4** **A** Flowchart of CCs sample inclusion and exclusion criteria. **B** Flowchart of FF sample inclusion and exclusion criteria.
