# Supplementary material for: Metabolomic profiling delineates the role of adenosine in oocyte quality and embryonic development
Source: Cell Death Dis. 2026 Jun 9;17(1):724. doi: 10.1038/s41419-026-08886-9 (PMC13473481; doi:10.1038/s41419-026-08886-9)
Supplement: Supplementary file 6 [file 41419_2026_8886_MOESM6_ESM.docx]

**Supplementary Table 1. Clinical epidemiological information of participants who provided FF**

|  | All  (n=30) | Rate of high-quality embryo rate < 40 %  (n=15) | Rate of high-quality embryo rate ≥ 40 %  (n=15) | *P* value |
| --- | --- | --- | --- | --- |
| Female factors | | | | |
| Age (years) | 30.070 ± 0.673 | 30.000 ± 1.082 | 30.133 ± 0.839 | 0.923 |
| BMI (kg/m^2^) | 21.810 ± 0.503 | 21.833 ± 0.828 | 21.787 ± 0.604 | 0.964 |
| Basal serum FSH (mIU/ml) | 7.604 ± 0.601 | 7.372 ± 0.539 | 7.835 ± 1.094 | 0.707 |
| AMH (ng/ml) | 4.018 ± 0.471 | 4.865 ± 0.775 | 3.171 ± 0.465 | 0.071 |
| Male factors | | | | |
| Age (years) | 31.500 ± 0.924 | 32.800 ± 1.639 | 30.200 ± 0.782 | 0.163 |
| BMI (kg/m^2^) | 24.630 ± 0.536 | 25.080 ± 0.827 | 24.173 ± 0.690 | 0.407 |
| Semen volume (ml) | 3.849 ± 0.277 | 3.571 ± 0.481 | 4.127 ± 0.277 | 0.325 |
| Sperm density (* 10^6^/ml) | 142.400 ± 19.770 | 152.053 ± 30.490 | 132.754 ± 26.000 | 0.634 |
| Sperm motility (%) | 59.950 ± 3.749 | 58.590 ± 5.850 | 61.310 ± 4.873 | 0.723 |
| Sperm deformity (%) | 94.800 ± 2.799 | 91.590 ± 5.560 | 98.000 ± 25.470 | 0.260 |
| Sperm fragmentation rate (%) | 14.190 ± 2.596 | 17.440 ± 4.530 | 10.710 ± 2.136 | 0.200 |
| Laboratory factors | | | | |
| No. of oocyte retrieved | 15.470 ± 1.315 | 17.933 ± 1.999 | 13.000 ± 1.518 | 0.059 |
| Rate of fertilization (%) | 80.060 ± 3.244 | 85.575 ± 4.000 | 74.555 ± 4.800 | 0.090 |
| Rate of high-quality embryo (%) | 42.030 ± 4.675 | 22.482 ± 3.007 | 61.575 ± 5.189 | < 0.001*** |
