# Supplementary material for: Metabolomic profiling delineates the role of adenosine in oocyte quality and embryonic development
Source: Cell Death Dis. 2026 Jun 9;17(1):724. doi: 10.1038/s41419-026-08886-9 (PMC13473481; doi:10.1038/s41419-026-08886-9)
Supplement: Supplementary file 7 [file 41419_2026_8886_MOESM9_ESM.docx]

**Supplementary Table 4. The** **PCR primers of different gene expression used in this research**

| Gene | PCR primers (5’-3’) |
| --- | --- |
| *Rn18S* | F: GTAACCCGTTGAACCCCATT  R: CCATCCAATCGGTAGTAGCG |
| *Ent1* | F: TTCTCCAGCAGCAAGAGCC  R: CCGAGGACACATTCTGGGAC |
| *Ent2* | F: CCGGAAGATCTGGCTGACG  R: TCTGGCCACAGGAAGTAGGA |
| *Cx37* | F: CTTTCATTGGGGGAAAGGCG  R: CTGGGCACCTCTCTTTCACTT |
| *JNK* | F: AGCAGAAGCAAACGTGACAAC  R: GCTGCACACACTATTCCTTGAG |
| *CycB1* | F: AAGGTGCCTGTGTGTGAACC  R: GTCAGCCCCATCATCTGCG |
| *Cdc27* | F: TTCCTCGCAGAACGACTATATGC  R: CGGTAGTAACAGGTTGCCAGTA |
